# Crystallography, materials and computation

**DOI:** 10.1107/S2052252514014122

**Published:** 2014-06-24

**Authors:** C. Richard A. Catlow

**Affiliations:** a Department of Chemistry, University College London, 20 Gordon Street, London, WC1H 0AJ, United Kingdom

**Keywords:** computation, editorial, materials

## Abstract

The growing role of structural studies of materials and of computation in structural science is highlighted.

The birth of crystallography 100 years ago was in the determination of the structures of inorganic materials. And materials continue to pose some of the most fascinating challenges in our discipline. Moreover, structural studies in materials science and indeed in all areas are increasingly supported by computation which now permeates all aspects of crystallography.

Materials science is increasingly focused on function – on optimizing a property (or more commonly a range of properties) for particular applications. For example, applications in battery and fuel-cell materials require optimization of ionic transport properties while optimization of solar-energy materials requires ‘band-gap engineering’. If this type of optimization is not to be purely empirical, it requires detailed understanding of structure and the relation between structure and function – a relationship that can be probed now by computation which, for example, can show how structural modifications influence ionic transport mechanisms and energetics. Moreover, crystallographic studies can directly reveal and confirm transport mechanisms proposed by computation as in the elegant work of Nishimura and coworkers (2008 ▶) which revealed the diffusion path of Li^+^ ions in the topical olivine-structured lithium-ion conductor, LiFePO_4_, which had been predicted by the molecular dynamics studies of Islam *et al.* (2005 ▶) and which is illustrated below ▶.

Structural studies of materials have been transformed over the last decades by several technical developments. The ability of powder diffraction using synchrotron and neutron sources (and also more recently with laboratory-based diffractometers) when coupled with the Rietveld method has had an profound influence on materials chemistry enabling high-quality structural studies on complex and industrially important materials such as zeolites and other microporous materials for which single crystals are unavailable. Microcrystalline diffraction using synchrotron sources has also led to exciting new structural chemistry. The properties of functional materials are, however, often controlled by local structural features – defects or dopants – which may provide, for example, the active sites for a catalytic reaction. Here the combination of diffraction with local techniques is essential and extensive studies have been reported combining powder diffraction with XAFS to probe simultaneously both long-range and local structure in catalytic materials. Indeed, the growing capability to combine diffraction with a range of spectroscopic techniques is greatly advancing our ability to determine key physical and chemical functionalities of materials.

Time-resolved studies of solid-state reactions and catalytic processes are of growing importance, where rapid data collection is essential and where the combination of diffraction with spectroscopy and small-angle scattering can be of great value as in the important area of structural studies of crystal growth. Developments in sources will be essential and offer exciting opportunities in this field.

Turning to computation, we have already highlighted the role of computer modelling in probing structure-related properties, but computation also plays a very direct role in structure determination owing to the growing ability of a range of computational techniques in modelling and predicting the structures of both crystalline and amorphous systems. Indeed structure prediction has long been a challenge in computational solid-state science – a challenge that was crystallized by the celebrated article of John Maddox in which he threw out the provocative statement:[Chem scheme1] 
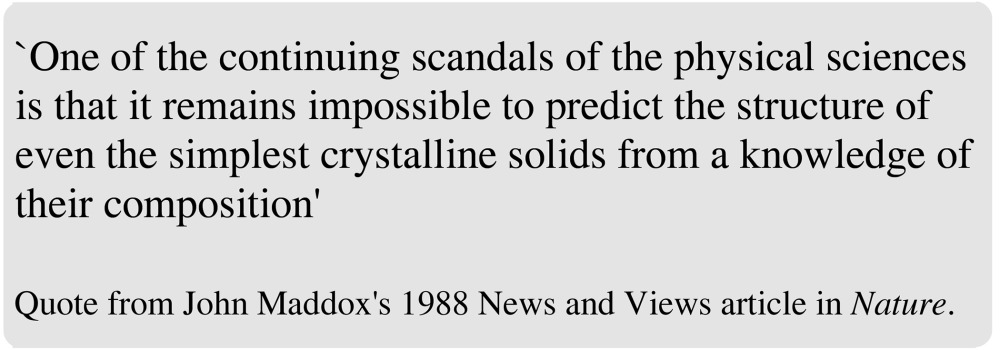


We can debate the accuracy of this statement when it was first made 25 years ago but, although structure prediction remains a real challenge, there has been enormous progress in recent years. It is now routine to refine approximate structures using minimization procedures applied to energies or perhaps free energies obtained from methods based on interatomic potentials or *via* an electronic structure calculation. Genuine prediction is much more difficult; and, as we have discussed elsewhere (Woodley & Catlow, 2008 ▶), the core of the problem is the development of effective ways of navigating the conformational space defined by all the structural variables to find those regions which could correspond to a plausible structure. Once identified, the energy or perhaps free-energy minimum of the region can readily be identified. An increasingly powerful range of methods are available for the initial search including techniques based on genetic algorithms, simulated annealing, molecular packing algorithms for molecular crystals, while for framework structured materials such as zeolites, topological approaches, of which there is a long history in crystallography, remain an intriguing and effective approach. Structure prediction is of course a major area of computational bio-molecular science and the field overall could profit from increased interaction between materials and bio-molecular developments and applications.

Computation can generate accurate models not only for crystallographic structures, but also for electronic structure; and there has been an explosion in recent years in electronic structure techniques, especially those based on density functional theory (DFT) which allow calculations on increasingly large systems at a level of accuracy that is acceptable for many applications. It is now a matter of routine to calculate electron-density maps for complex molecules and solids which can assist and amplify the interpretation of experimental data. Other properties may also be calculated, including elastic and dielectric constants. Moreover, computation methods are not restricted to perfect bulk properties, but can model point and extended defects and surfaces. Indeed computational modelling is now deeply embedded in the fields of both the defective solid-state and surface science. Moreover, the horizons of the field will continue to grow with developments in technique and the on-going growth in computer power.

How will these two closely related areas of structural science continue to develop? There is no doubt that new sources, technologies and techniques will extend our ability to probe complex structural problems and to elucidate further the ways in which structural modification affects function. Computation will acquire both increasing predictive power and a growing capability to model real complex materials. Materials science and computation will be increasingly interwoven and will continue to provide exciting and important challenges for crystallographic science. We encourage you to report and record your best work in this field in **IUCrJ**, which plans to give in-depth coverage to the materials and computation area.

## Figures and Tables

**Figure 1:**
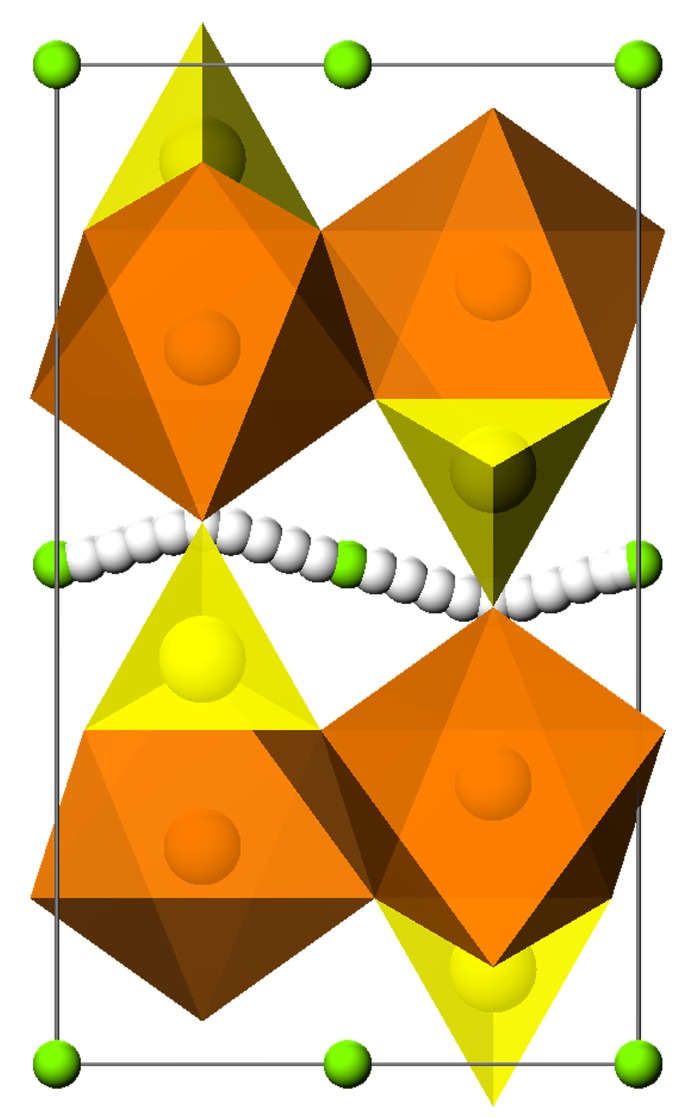
Path for lithium-ion migration in LiFePO_4_, as predicted by simulations of Islam *et al.* (2005 ▶). Reprinted with permission. Copyright 2005 American Chemical Society.
